# Supplementary material for: DMRforPairs: identifying Differentially Methylated Regions between unique samples using array based methylation profiles
Source: BMC Bioinformatics. 2014 May 15;15:141. doi: 10.1186/1471-2105-15-141 (PMC4046028; doi:10.1186/1471-2105-15-141)
Supplement: Additional file 2 — DMRforPairs output for the comparison of A431-MCF7 and NA17018-NA17105. Please start from the HTML files in each folder. Available via the BMC Bioinformatics website. [file 1471-2105-15-141-S2.zip › 1394847754114233_MOESM2_ESM/A431_MCF7/figures/21150.pdf]

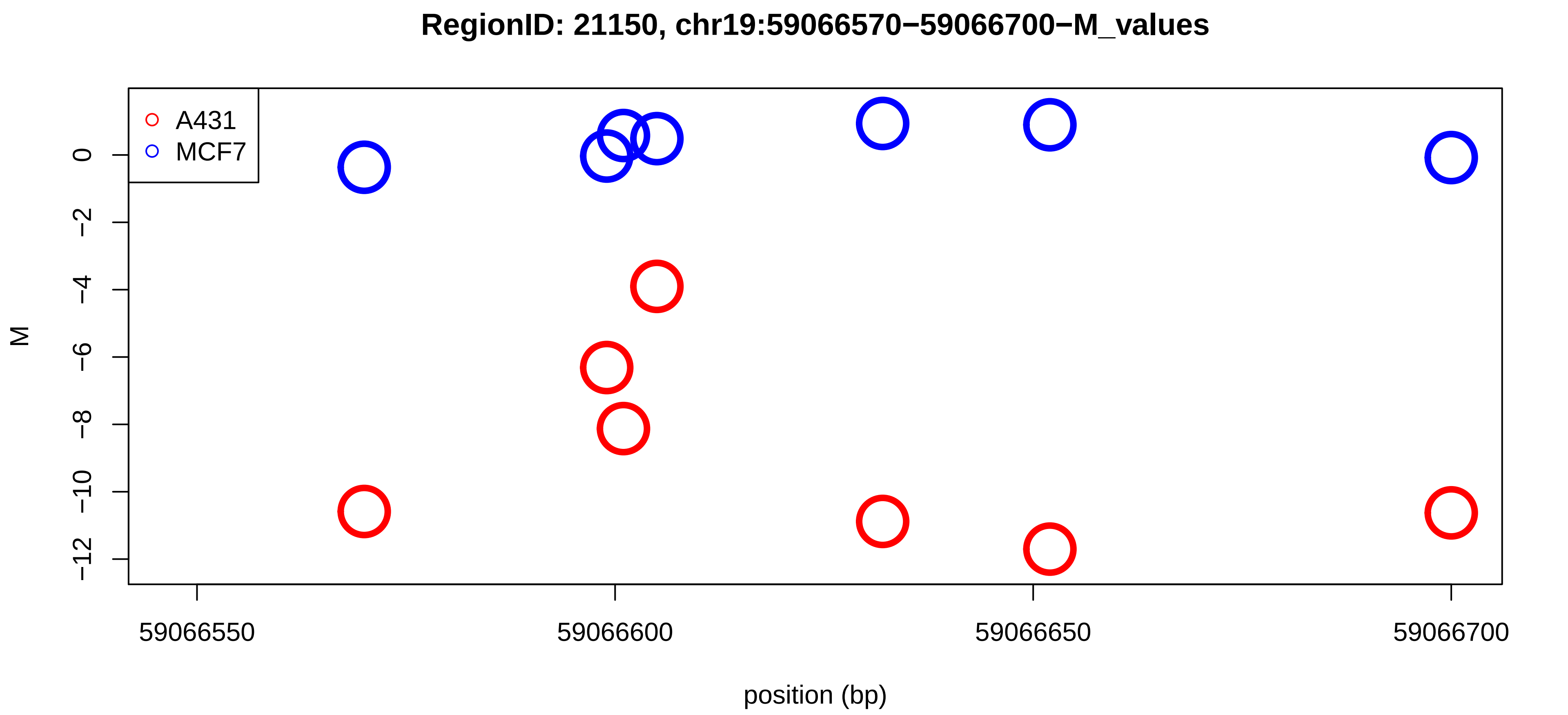

RegionID: 21150, chr19:59066570−59066700−M_values
A431
MCF7
M
0
−2
−4
−6
−8
−10
−12
59066550
59066600
59066650
59066700
position (bp)

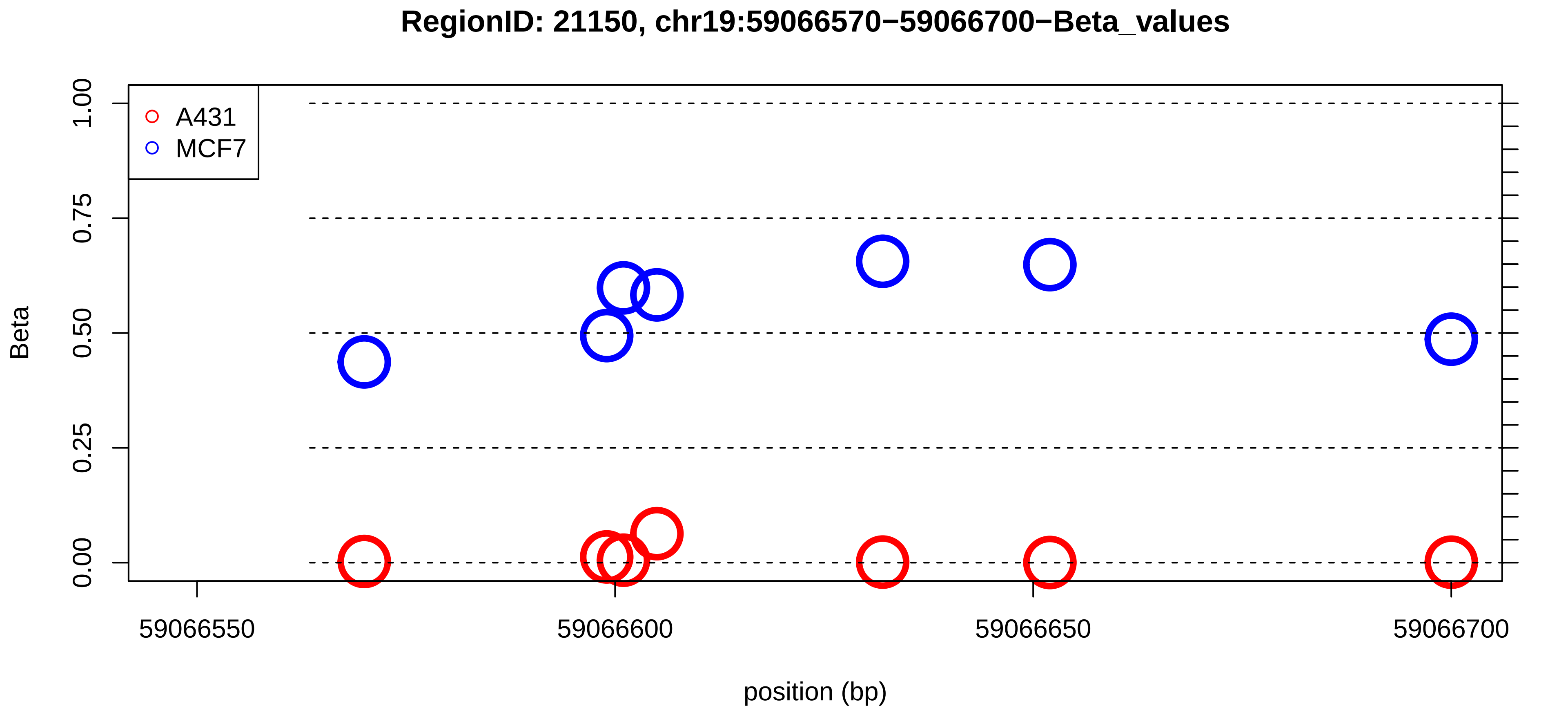

RegionID: 21150, chr19:59066570−59066700−Beta_values
A431
MCF7
Beta
1.00
0.75
0.50
0.25
0.00
59066550
59066600
59066650
59066700
position (bp)

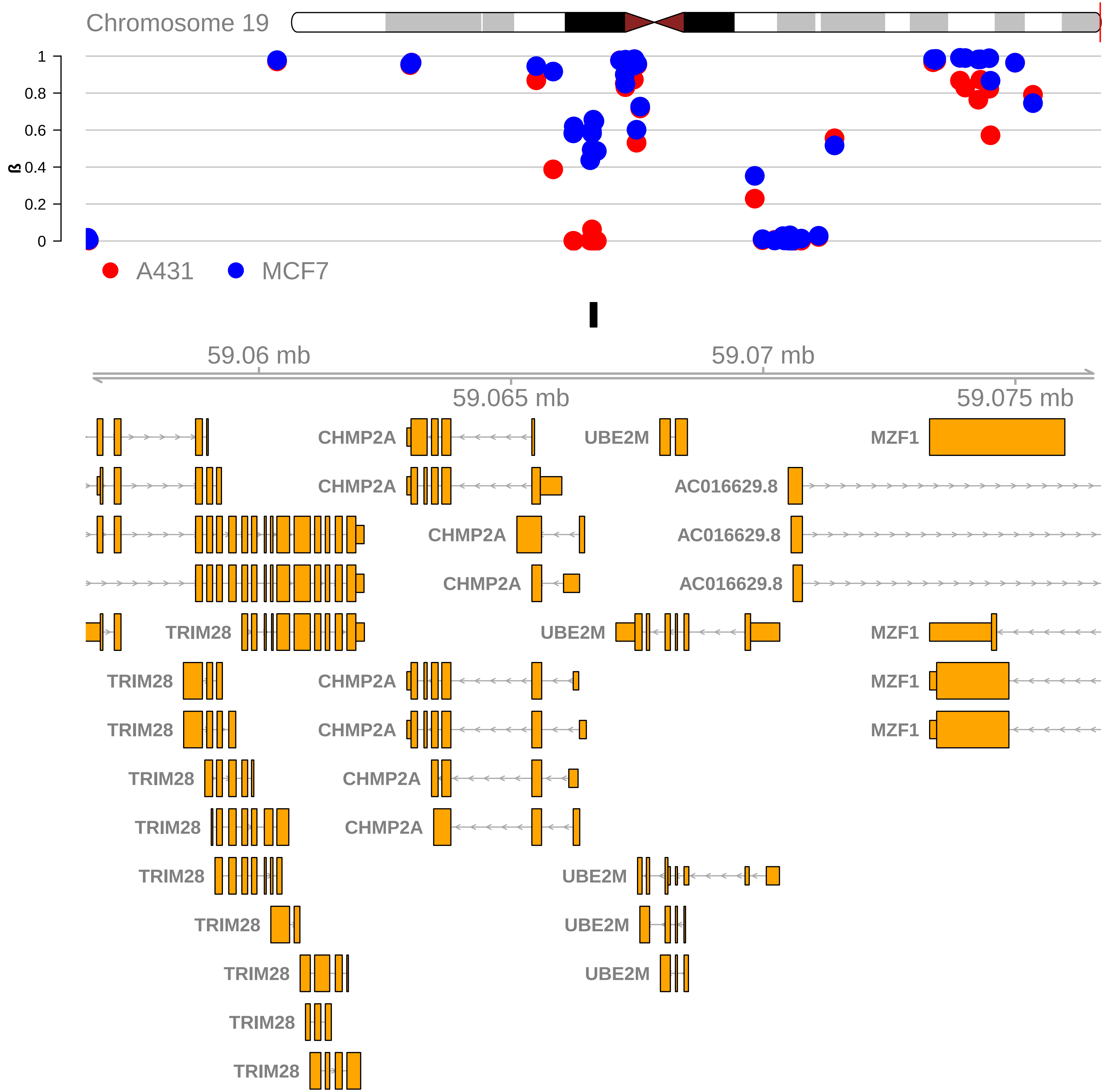

Chromosome 19
1
0.8
0.6
ß 0.4
0.2
0
A431
MCF7
59.06 mb
59.07 mb
59.065 mb
59.075 mb
CHMP2A
UBE2M
MZF1
CHMP2A
AC016629.8
CHMP2A
AC016629.8
CHMP2A
AC016629.8
TRIM28
UBE2M
MZF1
TRIM28
CHMP2A
MZF1
TRIM28
CHMP2A
MZF1
TRIM28
CHMP2A
TRIM28
CHMP2A
TRIM28
UBE2M
TRIM28
UBE2M
TRIM28
UBE2M
TRIM28
TRIM28
